# Traffic Management for Emergency Vehicle Priority Based on Visual Sensing

**DOI:** 10.3390/s16111892

**Published:** 2016-11-10

**Authors:** Kapileswar Nellore, Gerhard P. Hancke

**Affiliations:** 1 Department of Electrical, Electronic and Computer Engineering, University of Pretoria, Pretoria 0028, South Africa; ghancke@ieee.org; 2 Department of Computer Science, City University of Hong Kong, Hong Kong, China

**Keywords:** VANETs, audio visual sensing, emergency vehicle, traffic lights, traffic monitoring, priority, distance measurement techniques

## Abstract

Vehicular traffic is endlessly increasing everywhere in the world and can cause terrible traffic congestion at intersections. Most of the traffic lights today feature a fixed green light sequence, therefore the green light sequence is determined without taking the presence of the emergency vehicles into account. Therefore, emergency vehicles such as ambulances, police cars, fire engines, etc. stuck in a traffic jam and delayed in reaching their destination can lead to loss of property and valuable lives. This paper presents an approach to schedule emergency vehicles in traffic. The approach combines the measurement of the distance between the emergency vehicle and an intersection using visual sensing methods, vehicle counting and time sensitive alert transmission within the sensor network. The distance between the emergency vehicle and the intersection is calculated for comparison using Euclidean distance, Manhattan distance and Canberra distance techniques. The experimental results have shown that the Euclidean distance outperforms other distance measurement techniques. Along with visual sensing techniques to collect emergency vehicle information, it is very important to have a Medium Access Control (MAC) protocol to deliver the emergency vehicle information to the Traffic Management Center (TMC) with less delay. Then only the emergency vehicle is quickly served and can reach the destination in time. In this paper, we have also investigated the MAC layer in WSNs to prioritize the emergency vehicle data and to reduce the transmission delay for emergency messages. We have modified the medium access procedure used in standard IEEE 802.11p with PE-MAC protocol, which is a new back off selection and contention window adjustment scheme to achieve low broadcast delay for emergency messages. A VANET model for the UTMS is developed and simulated in NS-2. The performance of the standard IEEE 802.11p and the proposed PE-MAC is analysed in detail. The NS-2 simulation results have shown that the PE-MAC outperforms the IEEE 802.11p in terms of average end-to-end delay, throughput and energy consumption. The performance evaluation results have proven that the proposed PE-MAC prioritizes the emergency vehicle data and delivers the emergency messages to the TMC with less delay compared to the IEEE 802.11p. The transmission delay of the proposed PE-MAC is also compared with the standard IEEE 802.15.4, and Enhanced Back-off Selection scheme for IEEE 802.15.4 protocol [EBSS, an existing protocol to ensure fast transmission of the detected events on the road towards the TMC] and the comparative results have proven the effectiveness of the PE-MAC over them. Furthermore, this research work will provide an insight into the design of an intelligent urban traffic management system for the effective management of emergency vehicles and will help to save lives and property.

## 1. Introduction

**Figure 1:**
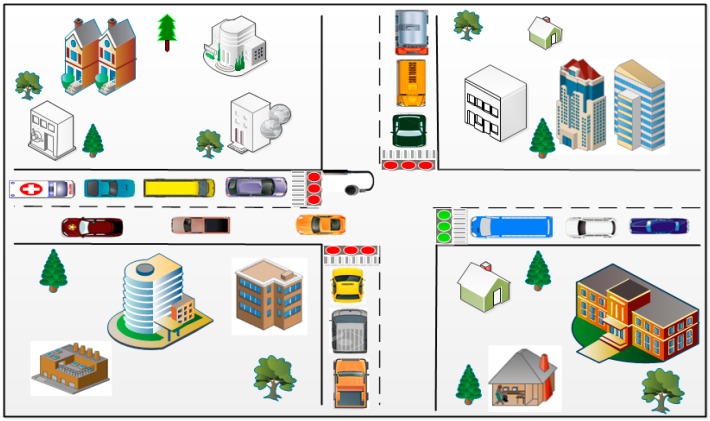
Emergency vehicle waiting at an intersection.

The traffic light control plays a vital role in any intelligent traffic management system. The green light sequence and green light duration are the two key aspects to be considered in traffic light control. In many countries, most traffic lights feature fixed sequences and light length duration. Fixed control methods are however only suitable for stable and regular traffic, but not for dynamic traffic situations. Looking at the present state of practice, the green light sequence is determined without taking the possible presence of emergency vehicles into account. Therefore, emergency vehicles such as ambulances, police cars, fire engines, etc. must wait in traffic at an intersection as depicted in Figure 1 which delays their arrival at their destination causing loss of lives and property. In Ireland, an average of 700 fatalities was noted every year due to late ambulance responses [1]. 

An increased volume of vehicles not only increases the response times of emergency vehicles, but it also increases the chances for them being involved in accidents. The emergency vehicle entering an intersection at a high speed on a red light poses danger to traffic on other roads and can cause accidents. The National Highway Traffic Safety Administration (NHTSA) has collected the ground ambulance crash data for the U.S between 1992 and 2011 (20 years) [2]. There were an estimated annual mean of 4500 motor vehicle traffic crashes and 1500 injury crashes involving an ambulance. In 20 years (1992–2011), 662 persons were killed and 52,000 persons were estimated to be injured in such crashes, including ambulance drivers, passengers, non-occupants and occupants of other vehicles. From the statistics of emergency vehicle accidents in U.S, there were roughly 31,600 accidents involving fire vehicles resulted in 645 fatalities over a 10 year period (2000–2009) and 300 fatalities that occur every year during police pursuits [3]. From the current problem section, it can be understood that, there is a serious need for an intelligent traffic management system for the effective management of both the normal and emergency vehicles. 

Many researchers have developed pre-emption systems that utilize the distance between the emergency vehicle and the intersection based on GPS for the signaling time. In this paper, we present a new approach to calculate the distance between the emergency vehicle and intersection using a real-time video feed from the cameras at intersections. An intelligent algorithm to shorten the travel time by scheduling both the green light sequence and green light duration based on the measured distance is also presented. A MAC protocol named PE-MAC is also proposed to deliver the emergency vehicle information to the TMC with less delay. A VANET model for the UTMS is developed, the NS is configured according to the proposed protocol and simulated in NS-2. We have planned to analyse and compare the performance of the PE-MAC with the standard IEEE 802.11p and other existing MAC protocols.

The remaining parts of this paper are structured as follows: Section 2 provides a review of related work. The UTMS architecture, functionality of TMC, distance measurement techniques and the proposed distance based algorithm are explained in detail in Section 3. The proposed PE-MAC and VANET model is presented in detail in Section 4. Experimental results are presented and discussed in Section 5. Section 6 concludes the paper.

## 2. Related Work

With an increasing amount of vehicles on the road, traffic congestion and transportation delays are increasing worldwide. Emergency vehicles, such as ambulances, fire engines and police cars, should be capable to react to emergency calls with minimum delay. The excellence of the emergency service depends on how fast the emergency vehicles can reach the incident location. If the emergency vehicle gets stuck in a traffic jam and its arrival at the incident location is delayed it can cause loss of lives and property. There is a need for smart traffic management systems based on priority and traffic density to improve the transportation efficiency and response times of emergency services.

Extensive work has been conducted on how to efficiently use traffic information to determine green light sequences. A number of traffic management schemes have been implemented to prioritize emergency vehicles [4]. Most of this research is associated with intelligent traffic control system design for providing clearance for emergency vehicles [5,6,7,8,9]. The traffic conditions are measured using cameras and the traffic parameters are estimated using the lane center edges [10]. An area-based image processing technique for the detection of traffic density was presented in [11].

Traditional traffic systems include strobe emitters, or light emitters that notice problems such as blocked line-of-sight and excessive noise [12]. Recent technologies such as infrared (IR) and Global Positioning System (GPS) have been used to detect emergency vehicle presence and calculate the real-time traffic density [13]. RFID tags have been used to identify the presence of emergency vehicles and the inductive loop method is used to count vehicles [14]. The widely used traffic detection methods, include video-based detection, microwave detection, radar detection, ultrasonic detection, etc. [15,16].

Wireless Sensor Networks (WSNs) allow for embedded sensors to be interconnected for observing and controlling consumer and industrial actions [17,18]. The use of Vehicular Sensor Networks (VSNs) or infrastructure WSNs have been proved to be promising solutions for monitoring and management of traffic. WSNs are flexible in terms of and energy efficiency and data collection type, e.g., video [19]. If a vehicle contains a WSN node, then localization algorithms [20,21,22] can be used to determine its location. The General Packet Radio Service (GPRS) technology can also be used for the dynamic control of traffic signals [23]. 

Nowadays, there are many emergency vehicle pre-emption (EVP) system designs including strobe light systems, infrared emitters, acoustic systems, and radio-based emitter/detector systems [24]. Usually, pre-emption works on the principle that an emergency vehicle is identified by sensors at each intersection and the traffic light controller switches ON the green light and holds it until the emergency vehicle exits the intersection [25]. The info-gap decision theory can also be used for actor coordination [26].

The green wave system, which provides a green wave to emergency vehicle by switching ON the green lights in the path of the emergency vehicle can be found in [27]. If the wave is disturbed, the vehicle queue in a green wave shoots up and may lead to over-saturation [28,29]. Technologies like RFID, Zig Bee, and Global System for Mobile communication (GSM) can be used for designing an intelligent traffic control system [30]. 

An RFID- and GPS-based automatic lane clearance system for ambulances is proposed in [31]. The main goal of this system is to reduce the travel time of an ambulance to the hospital by automatically clearing the lane, before the ambulance reaches the intersection. Vehicular Ad-Hoc Networks (VANETs) have been proven effective communication methods between an emergency vehicle and a traffic control system [32]. The connection admission control (CAC) algorithm has proven its better QOS and complexity performance [33]. Fuzzy control approaches have been adapted to monitor real-time traffic and handle the crowded traffic flow [34].

From the previous discussion, it can be understood that there are many techniques for providing clearance for emergency vehicles. Each of these techniques seems to have merits and demerits. In this research work an approach that combines the measurement of distance between the emergency vehicle and an intersection using visual sensing methods and PE-MAC for fast transmission of emergency vehicle information is proposed.

## 3. Proposed Methodology

In this section, we present the architecture of the urban traffic management system considered in our work, the functionality of the traffic management center, distance measurement techniques and a distance-based emergency vehicle dispatching algorithm. 

### 3.1. The Architecture of an Urban Traffic Management System (UTMS)

**Figure 2:**
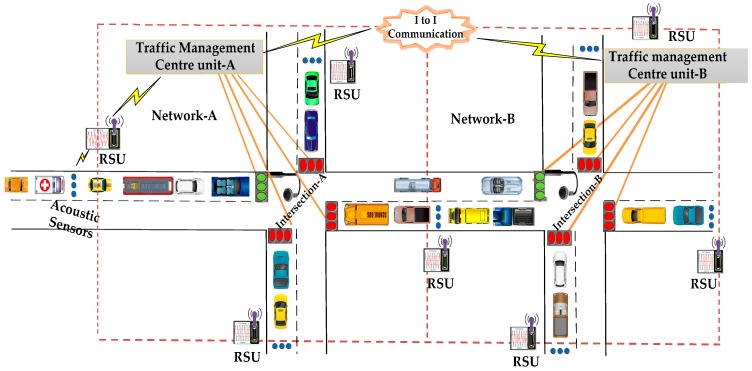
Architecture of an urban traffic management system.

WSNs and VANETs for smart cities [35] are becoming a reality with increased options for area coverage and connectivity stemming from machine-to-machine communication [36] and the Internet-of-Things [37,38,39,40,41]. An Urban Traffic Management System (UTMS), depicted in Figure 2, refers to a system that integrates sensing technologies, data processing techniques, wireless communications and advanced technologies to reduce traffic congestion, travel time, fuel consumption and provide priority-based signaling. On obtaining the data of emergency vehicles from sensors, the Traffic Management Centre (TMC) follows the distance-based emergency vehicle dispatching (DBEVD) algorithm and provides signals to the emergency vehicle immediately. The TMC of the present intersection (TMCA) provides the TMC of next intersection (TMCB) with the velocity of emergency vehicle and vehicle count moving towards intersection-B. As the TMCB knows the velocity of the emergency vehicle, it can estimate its arrival time at intersection-B. The TMCB determines the green light sequence and green light duration based on the estimated arrival time of the emergency vehicle, and the vehicle count value sent by the TMCA. Therefore, the emergency vehicles passes through the intersections with no or little delay. On using the proposed algorithm, the UTMS can effectively handle the emergency vehicles and thus save lives and property. 

### 3.2. Traffic Management Center

**Figure 3:**
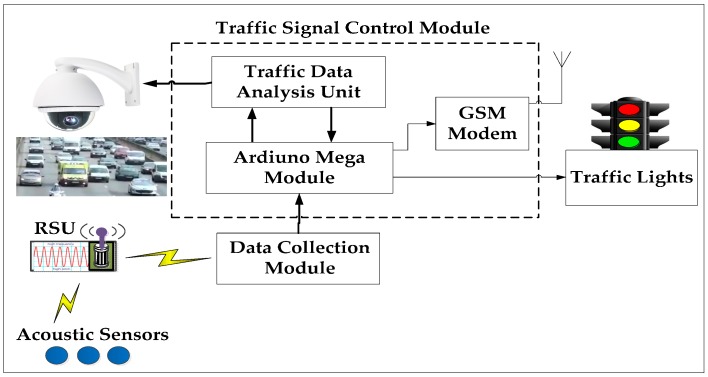
Schematic of a traffic management centre.

In this subsection, we describe the functionality of the Traffic Management Centre (TMC) and the role of each of its units. The schematic of a typical Traffic Management Centre (TMC) is presented in Figure 3. The presently used traffic light pre-emption systems can be categorised based on their operation as: optical systems, radio-controlled systems, GPS-based systems and acoustic sensor-based systems. Acoustic sensor-based systems outperform the other pre-emption systems in terms of accuracy and installation cost. Extensive work has been conducted on detecting emergency vehicles based on their siren sounds. We summarize the proposed approaches for emergency vehicle detection based on siren sounds in Table 1. 

The acoustic sensors collect the siren signals and forward them to the Road Side Unit (RSU). The Road Side Unit (RSU) includes a frequency measuring controller (Arduino UNO) to detect the emergency vehicles. The RSU collects the siren signals from the acoustic sensors and forwards them to the frequency measuring controller. The controller detects the emergency vehicle by its siren frequencies. The controller measures the frequencies of siren signals and computes the average of measured frequencies. The frequency measuring controller sends the alert signal to the traffic signal controller (Arduino Mega), if the frequency is between the range of yelp or wail. The traffic signal controller stops the fixed sequence and light length algorithm and executes the emergency vehicle dispatching algorithm on receipt of arriving emergency vehicle information. The data collection module gathers the data from all the RSU’s and forwards it to Traffic Signal Control Module (TSCM). The TSCM has two units, namely traffic analysis unit and traffic signal controller (Adriano mega). The camera sensor captures the real-time traffic video and inputs the traffic analysis unit, where the raw traffic data is processed and analyzed. The traffic controller unit gets the data like distance, velocity, traffic density, vehicle count, etc. from the traffic analysis unit. The controller executes the proposed algorithm and sends its decision to traffic lights. After the passage of an emergency vehicle, the system resumes its normal operation, i.e., fixed sequence and light length algorithm. In the following, we discuss distance measurement techniques, vehicle counting methods, a distance-based emergency vehicle dispatching algorithm and the simulation environment. 

### 3.3. Distance Measurement Techniques

At present, the popular distance measurement techniques include ultrasonic, infrared, laser, machine vision and radar measurements. The distance measurement based on machine vision obtains the value of the distance by the real-time processing of visual signals. There are different techniques to measure the distance between the vehicle and the camera. We perform the distance measurement by computing the Euclidian distance, Manhattan distance and Canberra distance [49,50]. In the following we briefly discuss these distances:

#### 3.3.1. Euclidean Distance 

It is the geometric distance in the multidimensional space. One technique that may suit a wide variety of image analysis applications is the distance transform or a Euclidean distance map. In general, if *a =* (*a*_1_, *a*_2_, *a*_3_,*…*, *a_m_*) and *b =* (*b*_1_, *b*_2_, *b*_3_,*…*, *b_m_*) are the two points in m-space, then the Euclidean distance (*d_ECD_*) between points *a* and *b* or *b* and *a* is as follows:

(1) dECD(a,b)=(b1−a1)2+(b2−a2)2+.........+(bm−am)2=∑i=1m(bi−ai)2

Let the pixels within a two-dimensional frame (*x*, *y*) be divided into two classes: object pixels and background pixels. For 2D points, *a =* (*a*_1_, *a*_2_) and *b =* (*b*_1_, *b*_2_) the Euclidean distance is:

(2) dECD(a,b)=(b1−a1)2+(b2−a2)2

#### 3.3.2. Manhattan Distance

The Manhattan distance between two points is the absolute sum of the differences of their coordinates. In general, if *a =* (*a*_1_, *a*_2_, *a*_3_,*…*, *a_m_*) and *b =* (*b*_1_, *b*_2_, *b*_3_,*…*, *b_m_*) are the two points in m-space, then the Manhattan distance (*d_MHD_*) between *a* and *b* is defined as follows:

(3) $$d_{MHD}(a,b)=\sum_{i=1}^{m}\left|a_{i}-b_{i}\right|$$

The Manhattan distance between points *a* = (*a*_1_, *a*_2_) and *b =* (*b*_1_, *b*_2_) is:

(4) $$d_{MHD}(a,b)=|a_{1}-b_{1}|+|a_{2}-b_{2}|$$

#### 3.3.3. Canberra Distance 

This distance is a weighted version of the Manhattan distance and is frequently used for data scattered around an origin. In general, if *a =* (*a*_1_, *a*_2_, *a*_3_,*…*, *a_m_*) and *b =* (*b*_1_, *b*_2_, *b*_3_,*…*, *b_m_*) are the two points in m-dimensional real vector space, the Canberra distance (*d_CAD_*) between *a* and *b* is given as follows:

(5) $$d_{CAD}(a,b)=\sum_{i=1}^{m}\frac{\left|a_{i}-b_{i}\right|}{\left|a_{i}|+|b_{i}\right|}$$

The Canberra distance (*d_CAD_*) between two vectors a, b in 2D real vector space is given by:

(6) $$d_{CAD}(a,b)=\frac{\left|a_{1}-b_{1}\right|}{\left|a_{1}|+|b_{1}\right|}+\frac{\left|a_{2}-b_{2}\right|}{\left|a_{2}|+|b_{2}\right|}$$

Since sensors’ performance in distance calculation is highly dependent on the environmental conditions, vision systems are highly preferred for moving vehicle distance calculations. The accuracy of the distance measurement techniques is computed by comparing the simulation results with the true practically measured distance.

### 3.4. Vehicle Counting Method

An input video sequence of road traffic can be processed and analyzed to get vehicle counts and speeds. The traffic management center can utilize this information in a traffic signal control module, resulting in an efficient traffic management. The vehicle counting method includes the following steps:

- Grayscale conversion.
- Foreground extraction.
- Defining Region of Interest (ROI).
- Thresholding.
- Filling the holes.
- Morphological operations.
- Detect the vehicles entering the ROI.
- Count the vehicles.

### 3.5. Distance Based Emergency Vehicle Dispatching Algorithm

**Figure 4:**
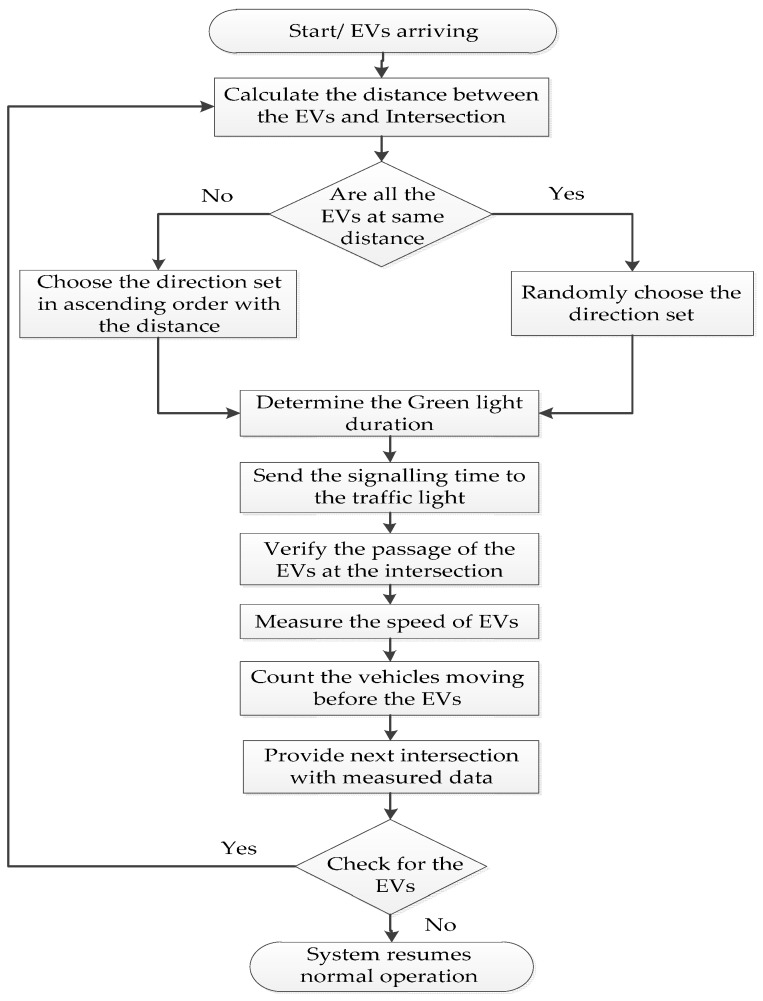
Distance-based emergency vehicle dispatching algorithm.

In order to achieve optimal traffic light control to provide clearance for any emergency vehicle and to shorten its travel time, we propose a distance-based emergency vehicle dispatching algorithm. We assumed only one emergency vehicle per direction. The proposed algorithm is represented in Figure 4. The EVs in the flowchart represent the emergency vehicles. The proposed algorithm has mainly six steps:

1. The sensor senses the presence of emergency vehicles. The emergency vehicles are ambulances, fire engines and police cars.
2. Calculate the distance between the emergency vehicle and the intersection.
3. The controller checks that the arriving emergency vehicles are at the same distance or not. If they are at the same distance, the controller randomly chooses the direction to set the green light. Else, he chooses the direction set in ascending order with the distance.
4. Determine the green light duration based on the measured distance values and send these values to the traffic lights.
5. Verify the passage of the emergency vehicle and measure the speed of the emergency vehicle and count the vehicles moving along with the emergency vehicle towards next intersection. The system sends the measured data to the next intersection.
6. The controller checks for the presence of the emergency vehicle. If no vehicle, then it resumes normal operation. Else, it continues repeats from step 2 to step 6.

In this research work, we focused on visual sensing methods for determining green light sequences and green light duration. Distance measurement techniques help us to find the nearest emergency vehicle to the intersection and determine the green light sequence.

Up to now, we have discussed in detail visual sensing methods to collect the emergency vehicle information. It is also important to talk about how fast the measured information is delivered to the TMC. For that we have investigated the Medium Access Control (MAC) layer in sensor networks to prioritize the emergency vehicle data and to reduce the transmission delay for emergency messages. We have modified the medium access procedure used in the IEEE 802.11p standard with the PE-MAC protocol, which is a new back-off selection and contention window adjustment scheme to achieve low broadcast delays for emergency messages. The PE-MAC is explained in detail in the next section.

## 4. PE-MAC Protocol

In this section, we present the Priority for Emergency Traffic based MAC (PE-MAC) Protocol, PE-MAC algorithm and the developed VANET model. 

### 4.1. Priority for Emergency Traffic Based MAC (PE-MAC) Protocol

**Figure 5:**
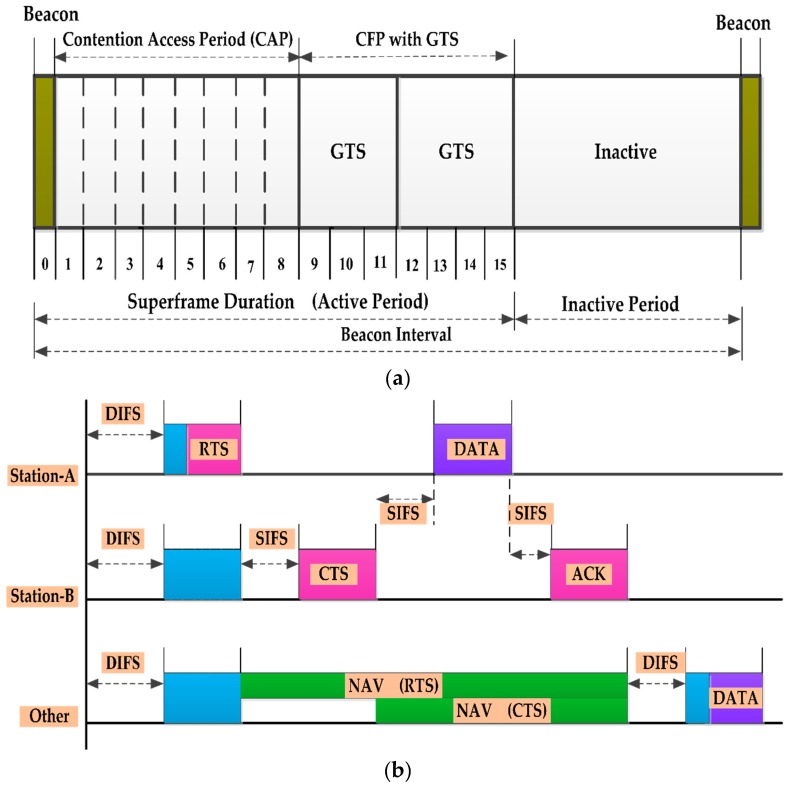
IEEE 802.11p (**a**) Superframe structure; (**b**) CSMA/CA process.

The IEEE 802.11p protocol has been developed by the IEEE 1609 working group as a key communication standard for vehicular networking. The IEEE 802.11p basically adopts the carrier sense multiple access with collision avoidance (CSMA/CA) with exponential back-off mechanism for packet access control. When a station wants to send a packet, first it has to listen to the channel, which is referred to as the carrier sensing. If the channel is free for a time known as the distributed inter frame spacing (DIFS) time, the station will transmit a request to send (RTS) to the destination. The destination will respond with a clear to send (CTS) if it is available to receive data. When the source station receives the CTS, it will transmit its data. The network allocation vector (NAV) indicates the time amount the channel is busy. All the packets sent in the network hold this NAV information. After the data has been correctly received at the destination station, it will send an acknowledgment (ACK) back to the sender station. At this point, if the sender has more data to transmit, it will again begin its back-off and repeat the process. The frame structure and the CSMA/CA process are demonstrated in Figure 5a,b, respectively. The short inter frame spacing (SIFS) is used as the wait time between the RTS, CTS, DATA and ACK frames. The SIFS ensures that the other node does not wrongly determine that the channel is idle during the handshake.

The main goal of our work is to provide priority to the emergency vehicle messages and to reduce their broadcast delay. To achieve this goal, we have planned to modify the medium access procedure used in IEEE 802.11p standard with the PE-MAC protocol, which is a new back-off selection and contention window adjustment scheme to achieve low broadcast delays. We have four different types of data: (1) ambulance data; (2) fire engine data; (3) police car data; and (4) normal vehicle data. The priority assignment to these four different types of data is given in Table 2. 

We have assumed a random variable “Y”, which represents the back-off delay value in the contention window interval [0, CW]. The back-off delay value (Y) can be defined as:

(7) $$Y/T\;=1\;\sim \;\mathrm{Normal}\;\mathrm{distribution}\;\left(\mu_{1}\;,{\sigma_{1}}^{2}\right)$$

(8) $$Y/T=2\;\sim \;\mathrm{Normal}\;\mathrm{distribution}\;\left(\mu_{2}\;,{\sigma_{2}}^{2}\right)$$

(9) $$Y/T=3\;\sim \;\mathrm{Normal}\;\mathrm{distribution}\;\left(\mu_{3}\;,{\sigma_{3}}^{2}\right)$$

(10) $$Y/T=4\;\sim \;\mathrm{Uniform}\;\mathrm{distribution}\;\left(\frac{3\mathrm{CW}}{4}\;,\;\mathrm{CW}\right)$$

where:

μ: Mean of the Normal Distribution, initialized with $\mu_{0}=\frac{CW}{8}$
σ: Variance of the Normal Distribution, such that $\sigma_{0}=\frac{CW}{8}$. Note that we set a fixed value of σ to allow many values of the back-off to be chosen.

**Figure 6:**
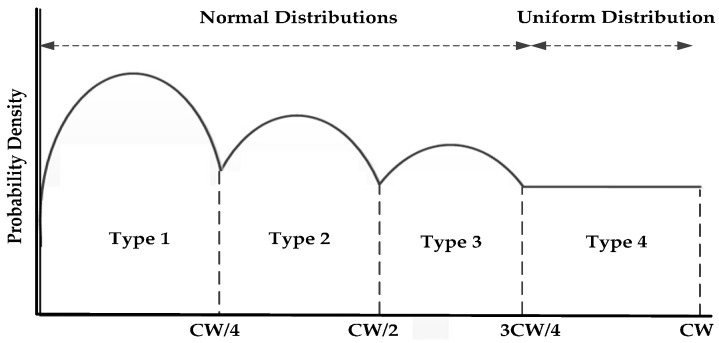
The back-off distribution used in PE-MAC.

A data type index T ∈ {1, 2, 3, 4} is attached to each packet. The back-off values are computed from the data index value (T), and the distribution as shown in Figure 6. A truncated normal distribution is used to ensure the positive back-off can only be positive. The normal distribution allows the node to pick a smaller back-off with high probability. We fix a smaller mean to draw the small back-off value. The mean values µ ∈ {µ_1_, µ_2_, µ_3_} are chosen like µ_1_ < µ_2_ < µ_3_ and there by the back-off values are B_OFF1_ < B_OFF2_ < B_OFF3_. The uniform distribution on the interval [3CW/4, CW] is used to draw the back-off values for the normal vehicles, hence ensuring lower delays for the ambulance than the other emergency vehicles and normal vehicles. 

The mean of the normal distribution is computed using the PE-MAC, Algorithm 1, which is explained later in this section. The main parameters that we have to talk about are delay threshold (D_Thr_), current MAC delay (D_K_) and current average MAC delay (D_K_ (Avg)). The delay threshold (D_Thr_), which is the maximum allowed delay per node for the transmission of emergency messages. The delay threshold can be computed by summing the DCF inter frame space time (TDIFS), short inter frame space time (TSIFS), estimated time to transmit a Request To Send (RTS) frame (TRTS), estimated time to transmit a Clear To Send (CTS) frame (TCTS), time to transmit a data frame (T_Data_), time to transmit an acknowledgement frame (Tack), and error tolerance value for the successful transmission (e_t_). The frame structure and the CSMA/CA are depicted in Figure. A node computes the MAC delay by subtracting the passing time, a packet is passed to the MAC layer from the sent time, a packet is actually sent onto the link. D_K_ and D_K-1_ are current MAC delay and stored previous average MAC delay. “α “ is a constant that determines the effect of the previous average MAC delay (D_K-1_ (Avg)) on the current average MAC delay (D_K_ (Avg)). The current average MAC delay can be written as:

(11) $$D_{K}\left(\mathrm{Avg}\right)=\left(1\;-\alpha \right)\times D_{K}\;+\;\alpha \times D_{K-1}\left(\mathrm{Avg}\right)$$

The PE-MAC algorithm prioritizes the emergency vehicle messages by dividing the contention window’s interval. The operation of the proposed algorithm is given in the following steps:

- Initialize all the variables.
- Compute the DThr and DK (Avg).
- Compare the DK (Avg) value with the DThr value. If the DK (Avg) ≤ DThr, then the mean value is assigned with the previous mean value and according to the type of messages, the contention window interval is divided. This is how a back-off value for the different messages is picked around the mean value.
- If DK (Avg) > DThr, the mean value is decremented so, that a smaller back-off value will be chosen in the successive iteration.

The proposed PE-MAC algorithm allows the emergency messages to be intimated to the TMC within the required and less delay.

**Algorithm 1.** Priority for Emergency Traffic based MAC (PE-MAC) **1:**
$\mu_{0}\leftarrow \frac{cw}{8}\;,\;\mu_{\mathrm{Thr}}\leftarrow \frac{cw}{16}\;,\;k\;\leftarrow 0$
**,**
**2:** Compute D_Thr_ and D_K_ (Avg)
**3:**    if (D_K_ (Avg) ≤ D_Thr_) then
**4:**  $\mu_{K+1}=\mu_{K}$
**5:**    **else**
**6:**  $\mu_{K+1}=\mu_{K}-0.5$
**7:**  **if**
$\mu_{K+1}\leq \mu_{\mathrm{Thr}}$
**8:**  $\mu_{K+1}=\mu_{0}$
**9:**  **end if**
**10: end if**
**11:** $CW_{T1}=\left[0\;,\mu_{K+1}+\;\sigma \;\right]$
**12:** $CW_{T2}=\left[\mu_{K+1\;}+\;\sigma \;,\;\mu_{K+1\;}+3\;\sigma \right]$
**13:** $CW_{T3\;}=\left[\mu_{K+1}+3\;\sigma \;,\;\mu_{K+1}+5\;\sigma \right]$
**14:** $CW_{T4\;}=\left[\mu_{K+1}+5\;\sigma \;,\;CW\right]$
This algorithm allows the emergency messages to be sent to the TMC within the required time and with less delay.

### 4.2. Simulation Environment

**Figure 7:**
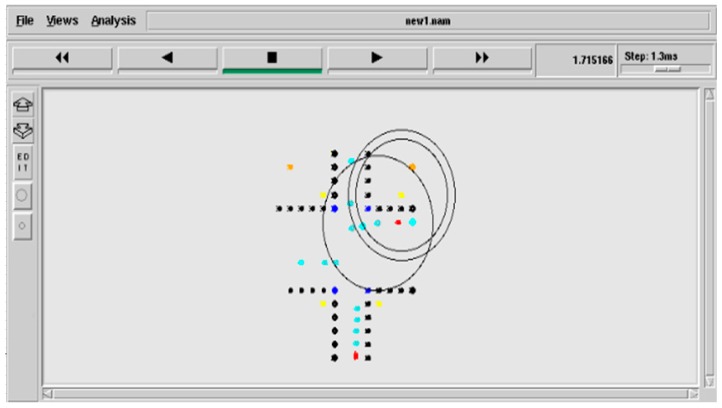
NAM of VANET simulation.

We wanted to examine the possibility of deploying an intelligent and dynamic traffic management system, which collects the priority information from the vehicles using visual sensing techniques, delivers the measured information to the TMC with less delay using the PE-MAC protocol and utilizes this information to effectively manage the traffic signals and thereby the emergency vehicles by developing a VANET model. A VANET model for the urban traffic management system shown in Figure 2 is developed and simulated in NS-2. The simulation parameters are given in the Table 3. Network animator (NAM) of the developed VANET simulation is shown in Figure 7. The black colour nodes represent the intersection, blue colour nodes represent the traffic signals, cyan colour nodes represent the normal vehicles, red coloured nodes represent the emergency vehicles, yellow colour nodes represent the RSUs and mustard colour nodes represent the TMCs (TMCA and TMCB). 

## 5. Results and Discussion 

In this section, the performance of the PE-MAC is evaluated and compared with the IEEE802.11p. The three performance metrics: the average end-to-end delay, throughput and residual energy are evaluated. We have also compared the average end-to-end delay of the proposed PE-MAC with IEEE 802.11p and IEEE 802.15.4 standards and the existing Enhanced Back-off Selection scheme for the IEEE 802.15.4 protocol [EBSS] [51]. We also present the simulation results of the vehicle counting method and distance measurement techniques.

### 5.1. Performance of the PE-MAC and IEEE802.11p

**Figure 8:**
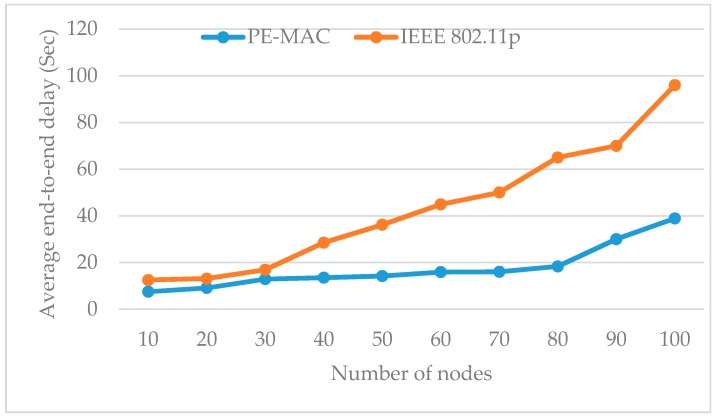
Impact of number of nodes on average end-to-end delay: proposed PE-MAC vs IEEE 802.11p.

The simulation of the VANET model for the urban traffic management system working with the proposed protocol is performed in NS2 with the number of nodes varying from 5 to 100. The simulation results show the performance of the PE-MAC and IEEE802.11p. The main parameters to be evaluated in this simulation are the average end-to-end delay, throughput and residual energy. The histogram plotted in Figure 8 compares the average end-to-end delay of the proposed PE-MAC and the IEEE 802.11p, protocol under varying number of node conditions. 

**Figure 9:**
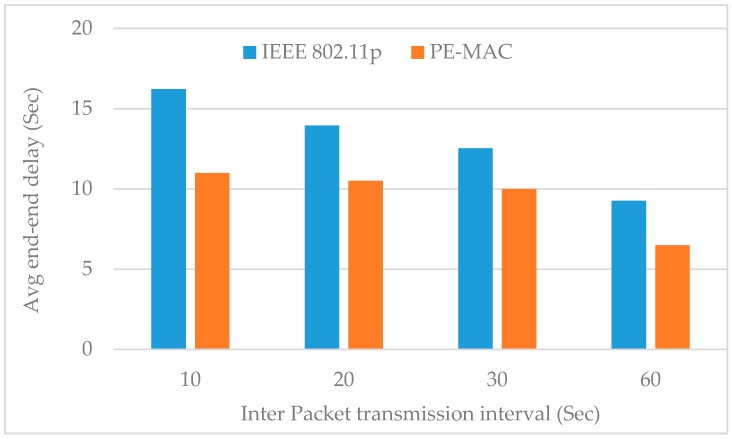
Impact of inter packet transmission interval on average end-to-end delay: proposed PE-MAC vs IEEE 802.11p.

The size of the network or the number of nodes shows its impact on the average end-to-end delay. The end-to-end delay increases with the node number. Despite this increase, it can be clearly observed from this figure that the proposed PE-MAC achieves a lower end-to-end delay compared to the IEEE 802.11p. When there are 10, 50 and 100 nodes in the network, the PE-MAC confirms the end-to-end delay of 15 s, 18 s and 40 s respectively, and the IEEE 802.11p achieves end-to-end delay of 17, 28 and 98 s respectively. The achieved improvement here is almost 11% at the number of nodes equal to 10 and increases to about 60% when the number of nodes equal to 100. Thereby, we confirm that the proposed PE-MAC delivers the information faster than the standard IEEE 802.11p.The histogram plotted in Figure 9 compares the average end-to-end delay of the proposed PE-MAC and the IEEE 802.11p, under various periodic packet transmission intervals. This figure clearly shows that the proposed PE-MAC achieves lower end-to-end delay compared to the standard IEEE 802.11p. The achieved improvement here is almost 28% in the case of the transmission interval of 10 s, and increases to about 42% when the transmission interval is 60 s.

**Figure 10:**
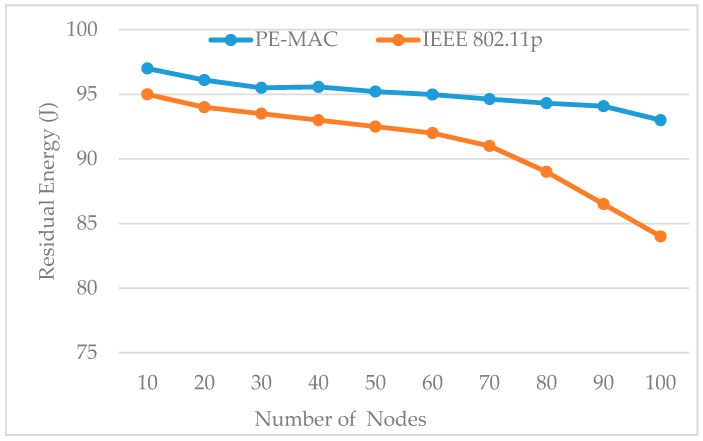
Impact of number of nodes on residual energy: proposed PE-MAC vs IEEE 802.11p.

Figure 10 shows the impact of the number of nodes on residual energy for both the proposed PE-MAC and IEEE 802.11p. The PE-MAC outperforms the IEEE 802.11p in terms of energy consumption. The residual energy of the PE-MAC is more compared to the IEEE 802.11p. As the node number increases, the intermediate nodes share the work and less energy is consumed for the transmission. These results have shown that the proposed scheme consumes less energy compared to the standard IEEE 802.11p. The initial energy we have taken is 100 J. The residual energy of the PE-MAC at the number of nodes equal to 10 is 98 J that means the energy consumed by PE-MAC is 2 J. The energy consumed by PE-MAC at 10, 50 and 100 number of nodes is 2, 5 and 8 J, respectively, and the energy consumed by IEEE 802.11p at 10, 50 and 100 number of nodes is 5, 8 and 16 J, respectively. The energy consumed by the proposed PE-MAC is almost 50% less than that of the energy consumed by the IEEE 802.11p protocol.

**Figure 11:**
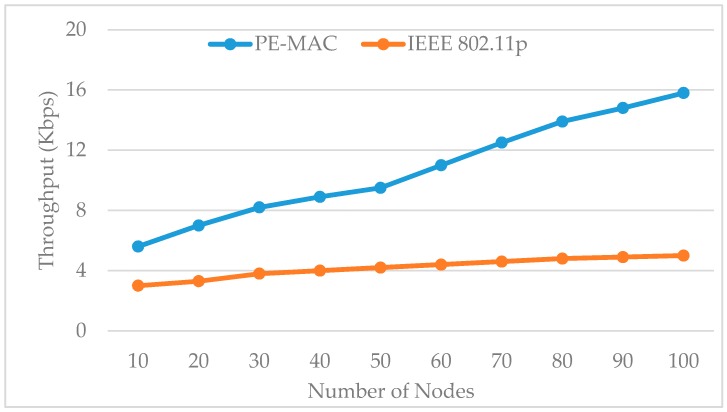
Impact of the number of nodes on throughput: proposed PE-MAC vs IEEE 802.11p.

Figure 11 shows the impact of the number of nodes on the throughput for both the IEEE 802.11p standard and the proposed PE-MAC. In comparison with IEEE 802.11p, PE-MAC keeps a better throughput for both the lesser and higher node number. When there are 20, 50 and 100 nodes in the network, the PE-MAC confirms the positive data delivery of about 7, 9.5 and 15.8 Kbps, respectively, and the positive data delivery of the IEEE 802.11p is 3.3, 4.2 and 5 Kbps, respectively. The achieved improvement here is almost 52% in the case the number of nodes equals 20, and increases to about 70% when the number of nodes equals 100. 

**Figure 12:**
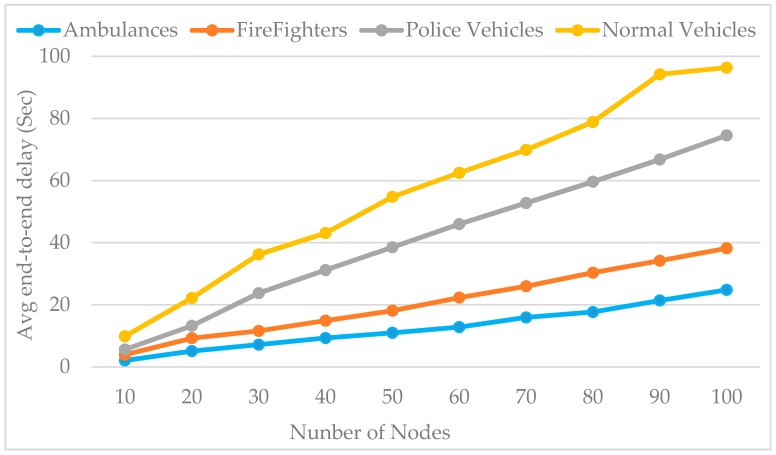
Impact of number of nodes on average end-to-end delay: all data type messages.

Figure 12 shows the impact of the network size on the average end-to-end delay of all the data type messages. We run our simulation with the PE-MAC and the results depicted in this figure clearly show that the end-to-end delay of the ambulance data is very small compared to the other emergency vehicles and normal vehicles. From these results, we can also observe that the average end-to-end delay increases with the network size. The results have proven that the proposed PE-MAC delivers the emergency messages to the TMC with less delay.

**Figure 13:**
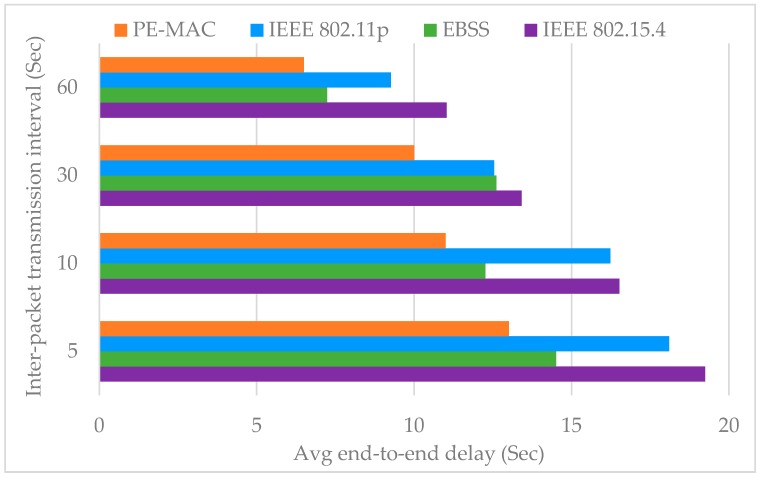
Impact of inter packet transmission interval on average end-to-end delay: for proposed PE-MAC, EBSS, standard IEEE 802.15.4 and IEEE 802.11p.

The histogram plotted in Figure 13 compares the average end-to-end delay of the proposed PE-MAC with standard IEEE 802.11p, IEEE 802.15.4 and the existing Enhanced Back-off Selection scheme for the IEEE 802.15.4 protocol [EBSS, an existing protocol to ensure fast transmission of the detected events on the road towards the TMC] [52], for various periodic packet transmission intervals. The achieved improvement here is almost 31% over IEEE 802.15.4, 11% over EBSS and 28% over IEEE 802.11p in the case of the transmission interval of 5 s, and increases to about 45% over IEEE 802.15.4, 16.6% over EBSS and 42% over IEEE 802.11p when the transmission interval is 60 s. The results have proven that the proposed PE-MAC achieves lower end-to-end delay compared to the other schemes considered, existing and standard.

### 5.2. Simulation Results of Vehicle Counting and Distance Measurement Techniques

In this subsection, we want to give the complete description of using visual sensing techniques to vehicle detection and counting. We collected the videos of road traffic with emergency vehicles using a digital camera setup. Five input videos of road traffic, around four to eight minutes were recorded. The spatial resolution of the recorded videos was 1920 × 1080 pixels, with temporal resolution of 30 frames per second. The vehicle counting method was implemented in MATLAB to count the vehicles moving along with the emergency vehicle towards next intersection.

**Figure 14:**
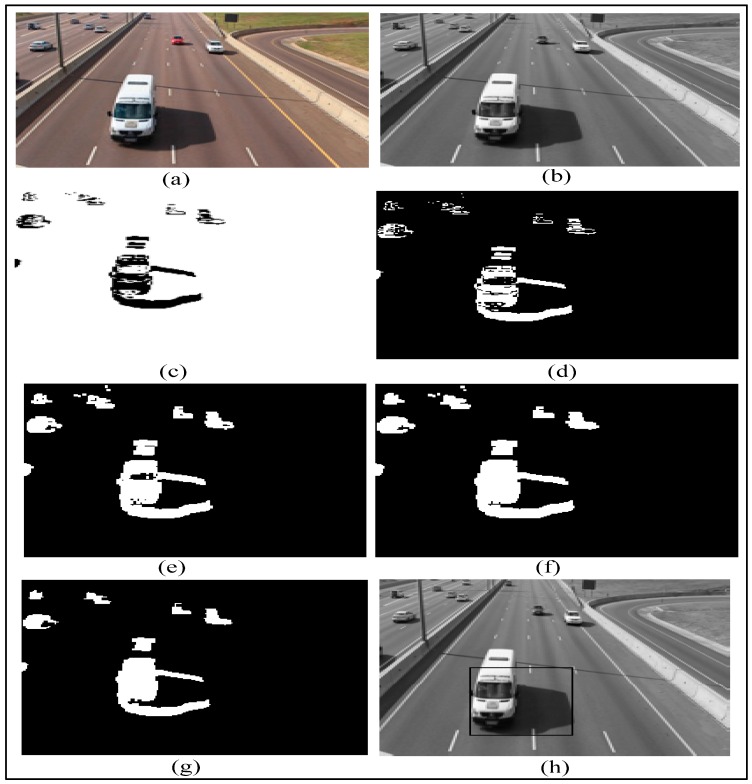
Emergency vehicle detection process: (**a**) RGB frame; (**b**) Grayscale frame; (**c**) Difference image; (**d**) Binary image; (**e**) Dilated image; (**f**) Hole filling image; (**g**) Eroded image; (**h**) Tagged Vehicle.

To achieve better results, the vehicle detection process should be done in the grayscale domain. Firstly a grayscale conversion is performed on each RGB video frame. The RGB frame and grayscale frame are shown in Figure 14a,b, respectively. Subsequently, we do foreground extraction by using the frame differencing technique. The difference image is shown in Figure 14c. The following step is to define a detection region. The subsequent step is to do thresholding, a method of image segmentation which separates the foreground pixels from the background pixels.

The thresholding operation results in binary image as shown in Figure 14d, which contains both false and missing foreground pixels. Hence, some more morphological techniques are used to eradicate noise and improve foreground objects. The morphological dilation is performed to recover some missing foreground pixels. The dilated image is shown in Figure 14e. The hole filling operation is performed to suppress black pixels surrounded by white regions. The resulting image after the hole filling operation is shown in Figure 14f. The morphological erosion operation will further convert the foreground items into the background. The result after a morphological erosion operation is shown in Figure 14g. After the morphological operations, the foreground items are found to be smoother and clearer. The detection zone defined previously is used to track and count the vehicles. When a vehicle enters the detection region, the bounding box appears around the moving vehicle as shown in Figure 14h.

Distance measurement plays an important role in determining the green light sequence. Distance measurement techniques help us find the nearest emergency vehicle to the intersection. We have measured the distance between the emergency vehicle and intersection using the Euclidean distance, Manhattan distance and Canberra distance techniques in MATLAB.

**Figure 15:**
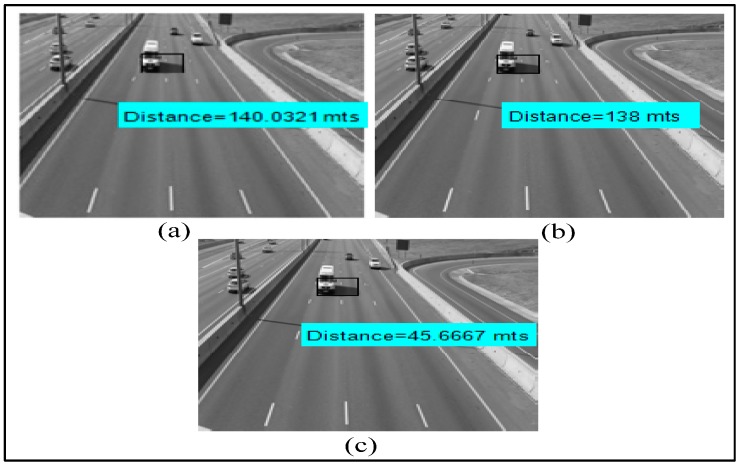
Distance measurement techniques: (**a**) Euclidean distance; (**b**) Manhattan distance; (**c**) Canberra distance.

Results of distance measurement using the Euclidean distance, Manhattan distance and Canberra distance techniques are shown in Figure 15a–c, respectively. We have done distance measurements at several points. The simulation values are compared with the true distance measured practically. The accuracy of each measurement technique is given in Table 4.

**Figure 16:**
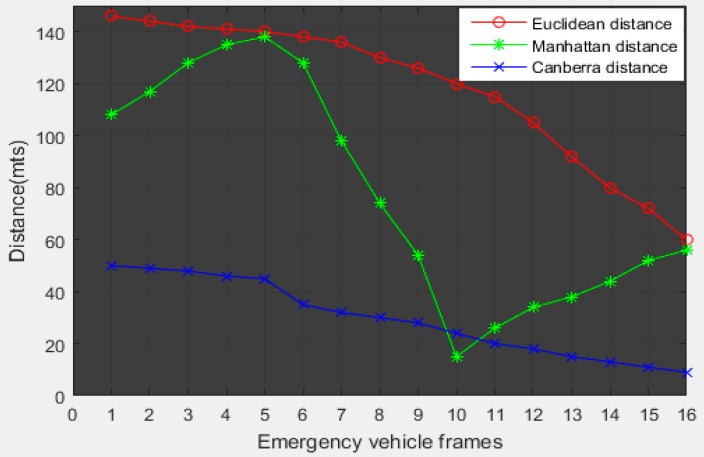
Comparison between the distance measurement techniques.

The simulation results show that the Euclidean distance outperforms other distance measurement techniques. Figure 16 gives the comparison between the distance measurement techniques. 

**Figure 17:**
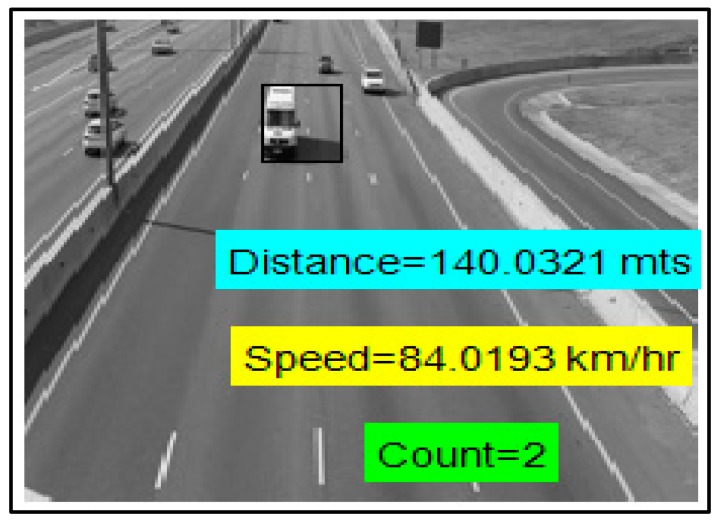
Measured data.

After obtaining the Euclidean distance, we measured the speed of the emergency vehicle (using ∆d/∆t) and counted the vehicles moving along with the emergency vehicle towards next intersection. The measured values of vehicle count, Euclidean distance and speed are shown in Figure 17. The traffic management center can utilize this information in a traffic signal control module, resulting in an efficient emergency traffic management process. All the existing works depend on some kind of infrastructure and require extra cost equipment. Our scheme utilizes ultrasonic sensors, RSUs and existing surveillance cameras. The image processing-based approach cuts the installation and maintenance costs compared to existing emergency vehicle pre-emption (EVP) systems [52,53]. 

## 6. Conclusions

This paper has presented an approach to schedule emergency vehicles in traffic. The approach combines the measurement of distance between the emergency vehicle and intersections using visual sensing methods, vehicle counting and time sensitive alert transmission within the sensor network. The distance between the emergency vehicle and the intersection is calculated from visual data using Euclidean distance, Manhattan distance and Canberra distance techniques for comparison. The experimental results have shown that the Euclidean distance outperforms other distance measurement techniques and is suitable for real-time applications. A complete description of the use of visual sensing techniques in vehicle detection and counting is also presented. The measured information like vehicle count, distance and speed are very useful for a traffic management center to manage emergency traffic efficiently. After obtaining the measured information, how fast it is delivered to the TMC is very important. For that, we have proposed a PE-MAC protocol, which is a new back off selection and contention window adjustment scheme to achieve low broadcast delay for emergency messages. A VANET model for the urban traffic management system used in this work is developed and simulated in NS-2. The NS-2 simulation results have shown that the PE-MAC outperforms the IEEE 802.11p in terms of average end-to-end delay, throughput and energy consumption. The performance evaluation results have proven that the proposed PE-MAC prioritizes the emergency vehicle data and delivers the emergency messages to the TMC with less delay compared to the IEEE 802.11p. The transmission delay of the proposed PE-MAC is also compared with the standard IEEE 802.15.4, and EBSS and the comparative results have proven the effectiveness of the PE-MAC over them. The improvement achieved by PE-MAC is almost 31% over IEEE 802.15.4, 11% over EBSS and 28% over IEEE 802.11p in the case of a transmission interval of 5 s, and increases to about 45% over IEEE 802.15.4, 16.6% over EBSS and 42% over IEEE 802.11p when the transmission interval is 60 s. The results have proven that the proposed PE-MAC achieves lower end-to-end delay compared to the considered schemes. From our work, we can confirm that the emergency vehicle information is measured accurately by using visual sensing methods and the measured information is delivered to the TMC in less time by adopting the proposed PE-MAC protocol. The potential impact of the proposed work is extensive as, being an inexpensive, effective, and accurate, it can be effectively applied in practice. Further research should be done on distance measurement in bad weather and high traffic conditions.

## Figures and Tables

**Table 1:** Approaches for emergency vehicle detection based on siren sounds.

Ref.	Proposed Approach	Outcome	Comments
[42]	Design and implementation of acoustic sensor-based automatic traffic control system.	Detects the emergency vehicle by their siren sounds.	The traffic light sequence is interrupted with the approaching emergency vehicle and thereby its waiting time at the intersection is reduced.
[43]	Low computational microcontroller-based siren sound detection system.	Designed a siren sound detection system with low processing power.	The proposed method outperforms the existing siren detection methods in terms of processing power and cost.
[44]	Detection of siren sounds using Fast Fourier Transform (FFT)	Detects the siren sound in 0 dB (S/N ratio). Detects the siren sound using the Doppler effect.	This work only detects the ambulance siren sound and neither alerts the traffic nor changes the traffic signals.
[45]	Detection of siren sounds based on a pitch detection algorithm.	Capable of detecting the emergency vehicle in the presence of pitched and non-pitched noise.	The proposed algorithm outperforms the complex pattern recognition algorithms. The siren signal miss rate of the algorithm is very low.
[46]	Emergency vehicle’s siren and flashing light detection based on acoustic and optical sensors.	Cost effective solution. Distinct emergency vehicles are detected.	The proposed system alert‘s the drivers of normal vehicles and pedestrians about the approaching emergency vehicle.
[47]	Cross microphone array-based emergency vehicle detection.	Determines the incoming direction of siren sound.	The proposed system for source detection outperforms the existing sound intensity techniques. It delivers precise warning data to the driver.
[48]	Digital image sensor-based emergency vehicle detection and display system for a vehicle.	Analyses and detects the emergency vehicle in an image using image processing techniques.	The proposed work alerts the driver when an emergency vehicle is detected. It is not cost effective as it needs the cameras to be mounted on the vehicle.

**Table 2:** Data types with priority assignment and access requirements.

Data Type, Index	Priority Assigned	Back-off Values	Medium Access Requirement
Ambulance data, 1	First priority (Highest)	B_OFF1_	Fast
Firefighter data, 2	Second priority	B_OFF2_ > B_OFF1_	Fast
Police car data, 3	Third priority	B_OFF3_ > B_OFF2_	Fast
Normal vehicle data, 4	Fourth priority (least)	B_OFF4_ > B_OFF3_	Fast or slow

**Table 3:** Simulation parameters.

Parameter	Value
Network Area	1500 m × 1500 m
Propagation model	Propagation/Two Ray ground
Network interface type	Physical/wirelessphy
Interface queue	Queue/Droptail/Priqueue
Channel type	Channel/Wireless channel
Antenna	Antenna/OmniAntenna
Visualization tool	NAM, Tracing
Routing protocol	DSR
MCA layer	IEEE 802.11p
Transmission rate	9.6 Kbps
Traffic type	CBN
Radio delay	10 m
Link layer type	LL
Packet size	512 bytes
IFQ length	50
Initial energy	100 J
No.of nodes	5 to 100
Speed	5, 10, 15 and 25 m/s

**Table 4:** Experiment Results.

Distance Measurement Techniques	Distance Measurement at Discrete Points (All Distances Are in Meters)			Accuracy	Outcome
	P1	P2	P3		
	True Value: 142	True Value :121	True Value: 62		
**Euclidean Distance**	140.03	120.25	60.66	98.60%	The simulation values are always very nearer to true values.
**Manhattan Distance**	138	54.03	56.45	77.61%	Only at some points, the simulation values are nearer to true values.
**Canberra Distance**	45.66	28	19.25	28.78%	The simulation values are always distant from the true values.
